# Serum Amyloid A3 is required for normal lung development and survival following influenza infection

**DOI:** 10.1038/s41598-018-34901-x

**Published:** 2018-11-08

**Authors:** Jennifer L. Ather, Oliver Dienz, Jonathan E. Boyson, Vikas Anathy, Eyal Amiel, Matthew E. Poynter

**Affiliations:** 1 Vermont Lung Center, Division of Pulmonary Disease and Critical Care, Department of Medicine, University of Vermont, Burlington, VT 05405 USA; 2 Department of Surgery, University of Vermont, Burlington, VT 05405 USA; 3 Department of Pathology and Laboratory Medicine, University of Vermont, Burlington, VT 05405 USA; 4 Department of Biomedical and Health Sciences, University of Vermont, Burlington, VT 05405 USA

## Abstract

Serum amyloid A (SAA) proteins are a family of acute phase apolipoproteins implicated to directly modulate innate and adaptive immune responses. However, new studies comparing endogenous SAAs and recombinant forms of these proteins have questioned the function of SAA in inflammation and immunity. We generated SAA3 knockout mice to evaluate the contribution of SAA3 to lung development and immune-mediated lung disease. While SAA3 deficiency does not affect the generation of house dust mite-induced allergic asthma, mice lacking SAA3 develop adult-onset obesity, intrinsic airway hyperresponsiveness, increased inflammatory and fibrotic gene expression in the lung, and elevated levels of lung citrullinated proteins. Polyclonally stimulated CD4^+^ T cells from SAA3−/− mice exhibit impaired glycolytic activity, decreased T_H_2 and T_H_1 cytokine secretion, and elevated IL-17A production compared to wild type cells. Polyclonally stimulated CD8^+^ T cells from SAA3−/− mice also exhibit impaired glycolytic activity as well as a diminished capacity to produce IL-2 and IFNγ. Finally, SAA3−/− mice demonstrate increased mortality in response to H1N1 influenza infection, along with higher copy number of viral RNAs in the lung, a lack of CD8^+^ T cell IFNγ secretion, and decreased flu-specific antibodies. Our findings indicate that endogenous SAA3 regulates lung development and homeostasis, and is required for protection against H1N1 influenza infection.

## Introduction

Serum amyloid A (SAA) proteins were first characterized as components of amyloid fibril deposits in amyloidosis, and later as lipoproteins bound to HDL in human plasma^1–3^. Further study proved SAA to be a robust biomarker of inflammation and injury, with levels increasing over a 1000-fold as part of the acute phase response^4,5^. The SAA family includes 4 distinct isoforms. SAA1 and 2 are highly homologous and predominantly produced by the liver. SAA3, which shares only ~60% homology with SAA1 and 2, is an acutely expressed isoform produced in non-primate mammals^5,6^. SAA4, also called C-SAA, is constitutively expressed and does not increase in response to infection or injury^7^. SAA3 is the predominant isoform expressed in mouse epithelial and hematopoietic cells^8^, and our previous work has demonstrated a rapid increase in *Saa3* gene expression in the lung in response to a variety of stimuli that induce innate and adaptive immune responses^9,10^.

SAA can interact with lipophilic macromolecules, including bacterial lipoproteins^11^. In addition, SAA has also been reported to be a potent opsonin of Gram-negative bacteria^12,13^, an inhibitor of viral entry into cells^14–16^, and a chaperone for retinoic acid (RA)^17^. RA is critical not only to normal lung development^18^, but also in the generation of allergic inflammatory disease^19^. In fact, dietary RA treatment has been utilized to repair TGF-β-induced lung injury in rats^20^. Recently, we discovered that hematopoietic cells from SAA3−/− mice express lower levels of retinoic acid receptors (RARs) than their wild type littermates, which inhibits their ability to respond to RA^10^. As a chaperone for RA, SAA may therefore also have key homeostatic functions in lung development, lipid transport, and inflammation.

Dysfunctions in lipid metabolism are linked to obesity and obesity-related lung disease^21^. Obesity can lead to a variety of inflammatory complications and may predispose to intrinsic airways hyperresponsiveness^22^. Additionally, obesity leads to worsened responses to H1N1 influenza infection, with obesity correlating to influenza hospitalization and mortality^23^. Obese individuals demonstrate decreases in both memory CD4^+^ T cell function as well as effector CD8^+^ T cell function^23^. H1N1 influenza infection causes respiratory illness and lung injury that is associated with elevated levels of interleukin-17A (IL-17A)^24^. Inhibition of IL-17A signaling in mouse models of H1N1 influenza decreases neutrophil recruitment and ameliorates influenza-associated lung injury^25,26^. Clearance of H1N1 influenza models relies heavily on the production of interferons^27^, and there is evidence to suggest that IFNγ regulates virus-specific CD8^+^ T cell homeostasis in influenza infection^28^.

We have generated a mouse lacking SAA3^10^ that, instead of showing decreased proinflammatory responses, exhibits adult-onset obesity, abnormal lung development and intrinsic airway hyperresponsiveness. Additionally, lack of SAA3 leads to increased innate proinflammatory responses and altered CD4^+^ and CD8^+^ T cell cytokine expression. Ultimately, mice lacking SAA3 have decreased survival in response to H1N1 influenza infection.

## Results

### Serum amyloid A3 deficiency increases baseline levels of inflammatory gene expression in the lung and contributes to intrinsic airway hyperresponsiveness

**Figure 1:**
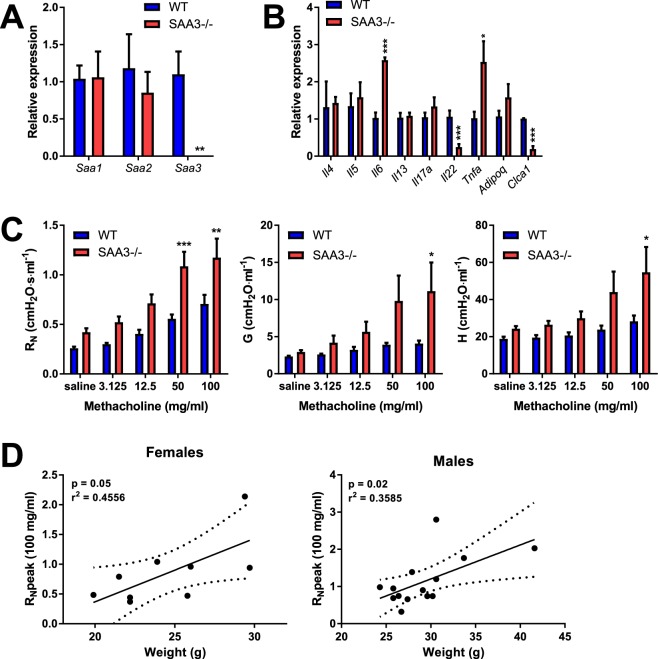
Characterization of SAA3−/− mice. Wild type and SAA3−/− littermates were analyzed at 18 weeks of age by Q-PCR for *Saa3* gene expression in lung. Characterization of different SAA isoforms was performed by Q-PCR from lung (**A**). The expression of a panel of genes was measured from lungs of WT and SAA3−/− mice (**B**). Pulmonary function assessment was analyzed by forced osciallation technique (**C**), and peak central airway resistance (R_N_) at the 100 mg/ml methacholine dose was plotted against body weight (D). n = 3–13/group. *p < 0.05, **p < 0.01, ***p < 0.005.

*Saa1* and *Saa2* demonstrated no compensatory increase in expression in the lung despite the absence of *Saa3* (Fig. 1A), however SAA3−/− mice expressed a differential inflammatory gene profile under basal conditions. SAA3−/− mice had higher expression of interleukin-6 (*Il6*) and tumor necrosis factor alpha (*Tnfa)* in the lung, while the expression of interleukin-22 (*Il22*) and the mucin-associated gene calcium-activated chloride channel 1 (*Clca1*) were significantly reduced in SAA3−/− mice compared to wild type littermates (Fig. 1B).

One prominent phenotype of our SAA3−/− mice, which we have recently explored in depth in a separate publication^10^, is the development of adult-onset obesity. As we and others have reported that obesity is associated with intrinsic airway hyperresponsiveness (reviewed in^21^), 18 week old wild type and SAA3−/− male and female mice were tested for methacholine hyperresponsiveness using the forced oscillation technique. As demonstrated in Fig. 1C, SAA3−/− mice had increased responsiveness to inhaled methacholine compared to wild type littermates, including exacerbated central airway resistance (R_N_), tissue resistance (G), and tissue damping (H). The exacerbated responses in R_N_ are significantly correlated to weight in both males and females (Fig. 1D), which raised the question of whether there are developmental differences in the lungs of the SAA3−/− mice that are independent of obesity.

### Mice lacking SAA3−/− develop maturation of airway smooth muscle and collagen deposition

**Figure 2:**
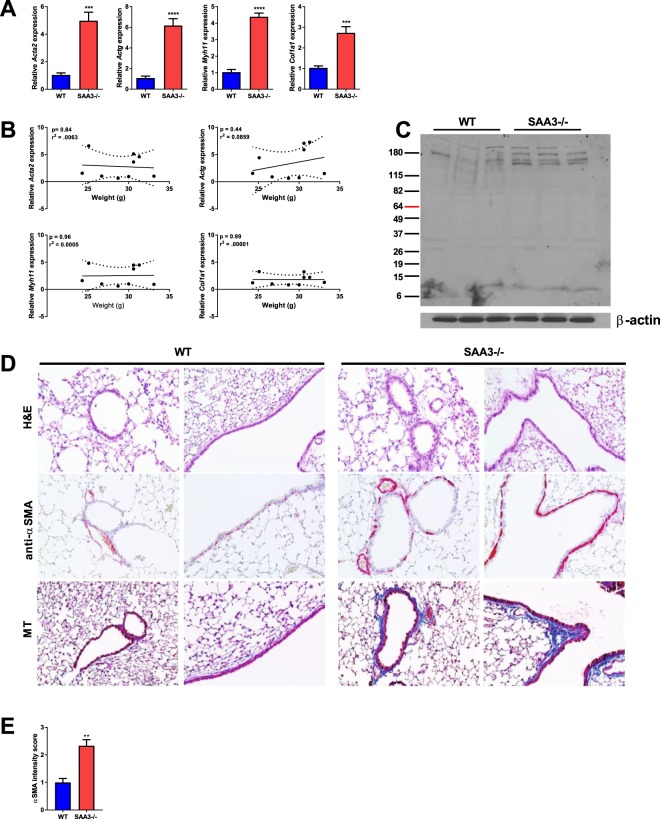
SAA3−/− mice exhibit augmented expression of airway smooth muscle and fibrotic genes in the lung. Lungs from 18 week old wild type (WT) and SAA3−/− mice were analyzed for gene expression by quantitative real-time PCR (**A**). Total body weight was plotted against fold expression gene changes (**B**). Lung lysates from 5 week old mice were analyzed for the presence of total citrullinated proteins and β-actin as a loading control by immunoblot (**C**). Formalin fixed lung tissue sections from 5 week old mice were stained with hematoxylin and eosin (H&E), anti-α-smooth muscle actin, and Masson’s Trichrome (MT) (**D**). Semi-quantitation of α-smooth muscle actin staining was performed by scoring of slides by three independent researchers blinded to the slide identities (E). n = 4–9/group. **p < 0.01, ***p < 0.005, ****p < 0.001.

To determine whether mice lacking SAA3 develop alterations in lung structure concurrent with methacholine hyperresponsiveness, wild type and SAA3−/− mice at 18 weeks of age were examined for the expression of genes involved in fibrosis and collagen deposition. SAA3−/− mice demonstrated significant increases in the gene expression of airway smooth muscle actin (*Acta2*), airway actin gamma (*Actg*), and myosin heavy chain 11 (*Myoh11*), indicative of a mature airway smooth muscle phenotype, as well as collagen 1a1 (*Col1a1*) (Fig. 2A). No changes were observed in vimentin or transgelin gene expression (data not shown). Gene expression was then plotted against body weight, and there was no significant correlation observed (Fig. 2B), indicating airway structural changes in these mice that are independent of obesity. Additionally, immunoblot analysis indicated the presence of increased citrullinated proteins in the lungs of very young (5 week old) SAA3−/− mice compared to wild type (Fig. 2C), and lung sections revealed an increase in staining for both alpha-smooth muscle actin (αSMA) and collagen in SAA3−/− mice compared to wild type littermates (Fig. 2D,E).

### The response to low-dose intranasal LPS is exacerbated in the absence of SAA3

**Figure 3:**
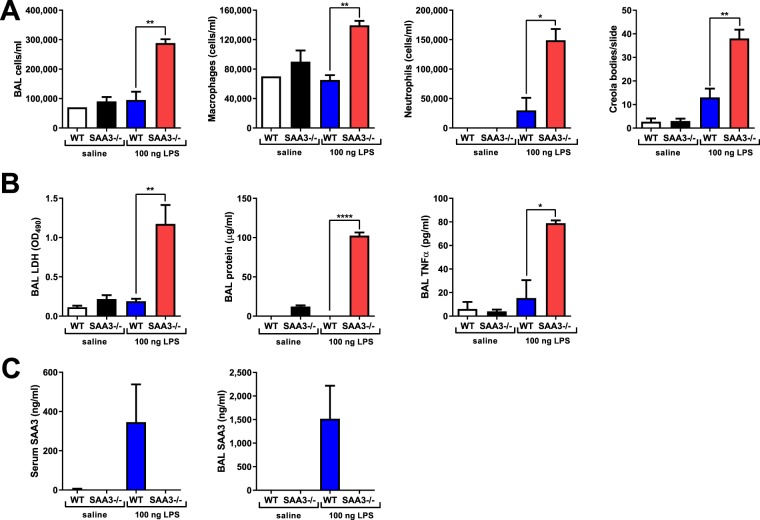
SAA3−/− mice exhibit an augmented response to intranasal LPS challenge. Wild type (WT) and SAA3−/− mice received 100 ng of LPS intranasally in 40 μl of sterile saline. Mice were analyzed 24 hours post challenge for BAL cellularity (**A**) and BAL LDH, total protein, and TNFα levels (**B**). SAA3 was measured from serum and BAL by ELISA (**C**). Data representative of two separate experiments. n = 3/group. *p < 0.05, **p < 0.01, ***p < 0.005.

It has been documented by our group and others^9,29,30^ that SAA3 is strongly induced in the lung in response to LPS inhalation, although the exact function of increased SAA3 in the LPS response is unknown. Wild type and SAA3−/− mice were administered a single intranasal instillation of a low dose of LPS (100 ng) in sterile saline and analyzed 24 hours later. This dose of LPS is frequently used to promote allergic sensitization to innocuous inhaled antigens and although not itself robustly inflammatory is reminiscent of the concentrations of LPS present in the homes of atopic children. SAA3−/− mice demonstrated increased cell recruitment into the lavageable airspaces, including increased macrophages and neutrophils compared to LPS-exposed wild type mice (Fig. 3A). In addition, cytospins from the BAL fluid revealed increased Creola bodies (sloughed epithelial cells) in the SAA3−/− mice (Fig. 3A). Further analysis of the BAL fluid indicated exacerbated release of lactate dehydrogenase and total protein into the lavageable airspaces, as well as a significant increase in the cytokine TNFα (Fig. 3B). Lack of SAA3 protein was confirmed in the serum and BAL fluid of SAA3−/− mice, whereas it was strongly induced by LPS instillation in the wild type mice (Fig. 3C).

### SAA3 is critical for CD4^+^ and CD8^+^ T cell function

**Figure 4:**
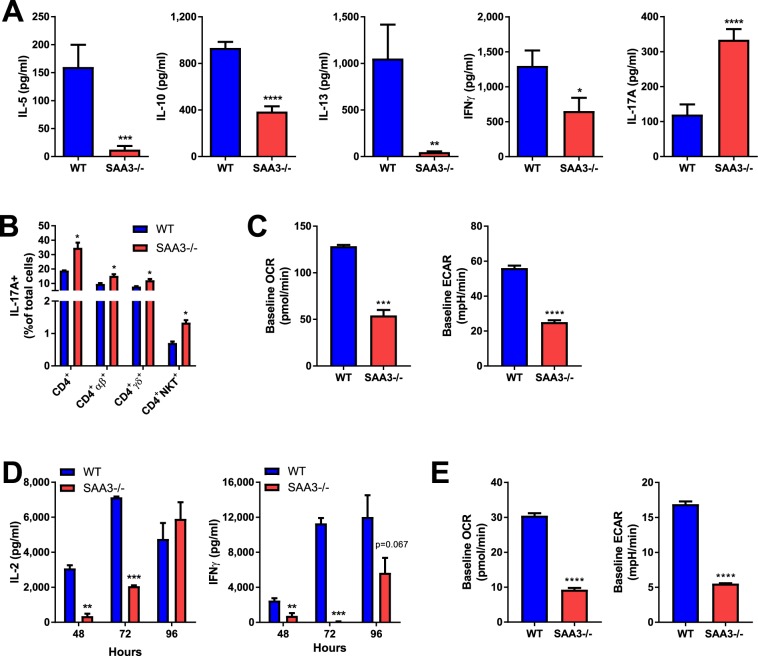
SAA3−/− mice exhibit altered T cell function. CD4^+^ T cells were isolated from the spleens and lymph nodes of WT and SAA3−/− mice and stimulated with anti-CD3/anti-CD28 for 96 hours. Cell supernatants were analyzed for cytokine content by ELISA (**A**). Cells were also collected for flow cytometry surface staining and intracellular staining for analysis of IL-17A^+^ cells (**B**). In separate experiments, polyclonally stimulated CD4^+^ T cells were analyzed by Seahorse for glycolytic activity (**C)**. CD8^+^ T cells were collected from spleen and lymph nodes, stimulated for 48, 72, and 96 hours. Supernatants were analyzed for cytokine content by ELISA (**D**). In separate experiments, polyclonally stimulated CD8^+^ T cell were analyzed by Seahorse for glycolytic activity (**E**). n = 7–18/group. *p < 0.05, **p < 0.01, ***p < 0.005, ****p < 0.001.

**Figure 5:**
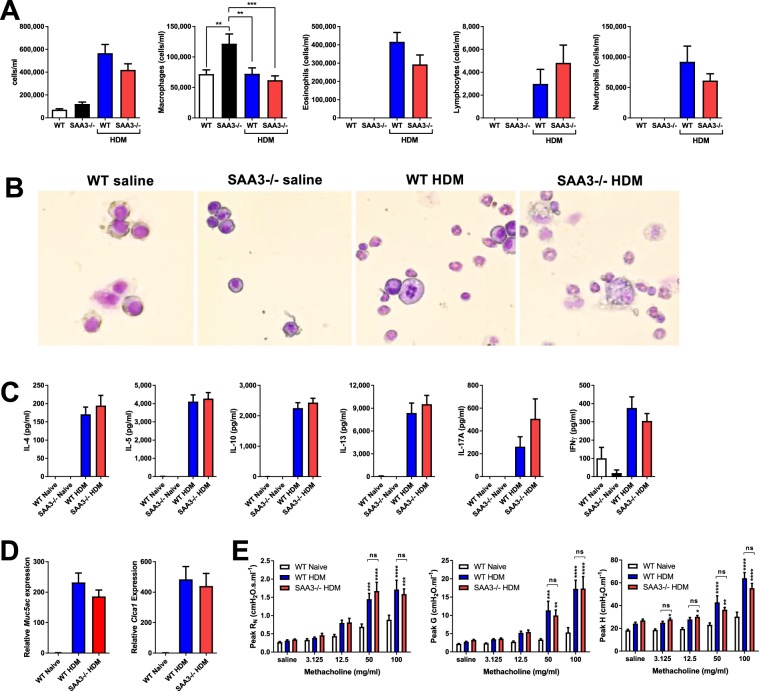
Mice lacking SAA3 respond comparably to wild type mice in a model of HDM-promoted allergic asthma. Wild type (WT) and SAA3−/− mice were sensitized and challenged to HDM and analyzed 24 hours after the final challenge. BAL cellularity was assessed by cytospin (**A**,**B**). Restimulated splenocytes were analyzed for cytokine production after 96 hours of restimulation (**C**). Whole lung expression of the mucin genes *Muc5ac* and *Clca1* were analyzed by Q-PCR (**D**). Pulmonary function assessment was analyzed by forced oscillation technique (**E**). n = 6–17/group. *p < 0.05, **p < 0.01, ***p < 0.005, ****p < 0.001.

To examine the impact of SAA3 deficiency on adaptive immunity, CD4^+^ T cells were isolated from the spleens and lymph nodes (a pool of mediastinal and inguinal lymph nodes) of naïve wild type and SAA3−/− littermates. CD4^+^ T cells were polyclonally stimulated for 96 hours with plate-bound anti-CD3 and soluble anti-CD28. Supernatants were collected and analyzed for cytokine secretion. Wild type CD4^+^ T cells effectively produced IL-5, IL-10, IL-13, IL-17A, and IFNγ (Fig. 4A, blue bars). In stark contrast, CD4^+^ T cells from SAA3−/− mice produced increased levels of IL-17A, while producing significantly less IL-5, IL-13, IL-10, and IFNγ (Fig. 4A, red bars). Intracellular flow cytometry analysis corroborated the increased presence of IL-17A-positive cells in the SAA3−/− CD4^+^ polyclonally stimulated cultures, including within the αβCD4^+^ and γδCD4^+^ subsets, as well as within the NKT subset (Fig. 4B). After 96 hours of polyclonal stimulation, these CD4^+^ cultures were analyzed by Seahorse extracellular flux analyzer for oxygen consumption rate (OCR, a proxy measure of mitochondrial respiration) and extracellular acidification rate (ECAR, a marker of glycolytic activity). SAA3−/− CD4^+^ cells exhibited decreased basal OCR and ECAR compared to wild type littermate controls (Fig. 4C). CD8^+^ T cells, also isolated from the spleens and lymph nodes of wild type and SAA3−/− mice, were polyclonally stimulated in the same manner for up to 96 hours. Wild type CD8^+^ T cells robustly produced IL-2 and IFNγ at all time points (Fig. 4D, blue bars), whereas SAA3−/− CD8^+^ T cells demonstrated a delayed and decreased production of both cytokines (Fig. 5D, red bars). Accordingly, SAA3−/− CD8^+^ T cells exhibited decreased basal respiratory and glycolytic function compared to wild type CD8^+^ T cells after polyclonal stimulation (Fig. 4E).

### Endogenous SAA3 does not affect the development of allergic airway responses in a house dust mite model of asthma

Given the effects of SAA3 deficiency on both innate and adaptive immune responses, as well as the intrinsic AHR present in these mice, we sought to assess the role of SAA3 in the development of the allergic airway response to house dust mite (HDM) extract. Wild type and SAA3−/− mice underwent inhalational allergen sensitization and challenge with HDM. All sensitized and challenged mice developed robust recruitment of inflammatory cells into the lavageable airspaces, including eosinophils, lymphocytes, and neutrophils, which were not significantly different between wild type and SAA3−/− mice (Fig. 5A,B). Splenocytes restimulated with HDM secreted the T_H_2 cytokines IL-4, IL-5, IL-10, IL-13, the T_H_17 cytokine IL-17A, and the T_H_1 cytokine IFNγ (Fig. 5C), and cytokine secretion was unchanged in SAA3−/− mice compared to wild type. Lung gene expression of the mucin genes *Muc5ac* and *Clca1* were robustly upregulated in both the wild type and SAA3−/− sensitized and challenged mouse groups (Fig. 5D), and methacholine responsiveness was likewise unaffected by the absence of SAA3 (Fig. 5E). The lack of a difference in response to the HDM model was surprising, based upon the intrinsic airway hyperresponsiveness and altered CD4^+^ T cell function we observed in our naïve and *in vitro* studies.

### SAA3 is critical for survival following influenza infection

**Figure 6:**
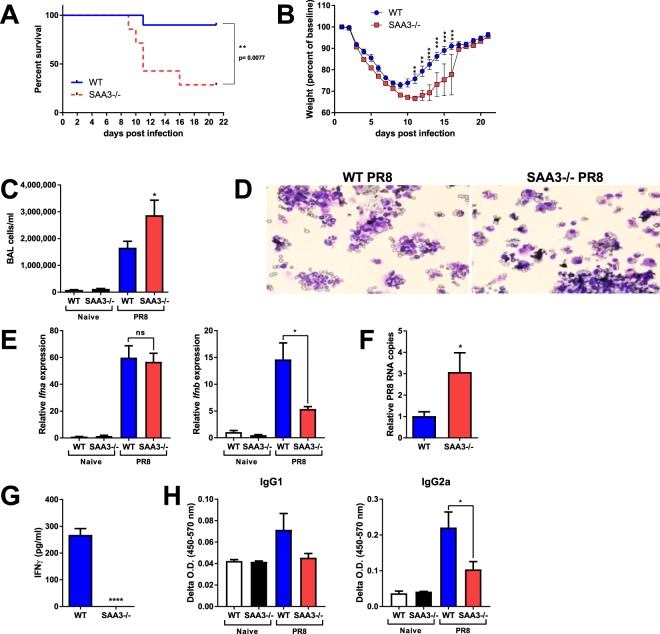
SAA3−/− mice succumb to influenza virus infection. Ten to twelve week old male mice were inoculated with 5000 EIU of PR8 H1N1 Influenza A and monitored for survival (**A**) and weight loss (**B**). Mice harvested at day 8 post inoculation were analyzed for BAL cellularity (**C**,**D**), *IFNa and IFNb* expression (**E**), and PR8 viral RNA copies from lung single cell suspensions (**F**). CD8^+^ T cells from the MLN of mice at day 8 were cultured in the presence of anti-CD3 for 96 hours and IFNy levels were measured from cell-free supernatants (**G**). Serum immunoglobulins were measured by ELISA (H). n = 8–10/group. *p < 0.05, **p < 0.01 ***p < 0.005 ****p < 0.001.

Considering their increased production of proinflammatory cytokines during LPS instillation and their apparent inability to mount an effective T cell response, we hypothesized that SAA3−/− mice could be more susceptible to infection. Therefore, ten week old male WT and SAA3−/− male mice were intranasally inoculated with 5000 EIU (egg-infectious units) of H1N1 Puerto Rico/8/34 (PR8) influenza A virus. Mice were monitored and declared moribund when they demonstrated labored breathing, little movement when startled, and greater than 30% weight loss. While all mice lost weight over the first ten days following PR8 influenza inoculation, SAA3−/− mice demonstrated significantly greater mortality than their wild type littermates (Fig. 6A), and of the surviving mice, the SAA3−/− mice were slower to regain their body weight (6B). In a separate experiment, 10 week old wild type and SAA3−/− mice mice were analyzed eight days after PR8 inoculation. Body weights did not differ between wild type and SAA3−/− mice prior to infection as a consequence of adult-onset weight gain (wildtype 27.55 ± 0.69 g, SAA3−/− 26.80 ± 1.05 g; p = 0.56) or following influenza infection (wildtype 21.24 ± 0.61 g, SAA3−/− 20.24 ± 0.77 g; p = 0.33). However, BAL fluid indicated an increased cellular recruitment in the SAA3−/− mice (Fig. 6C), predominantly neutrophils (Fig. 6D), which was accompanied by a decreased expression of *Ifnb* (Fig. 6E) and a greater abundance of PR8 RNA copies in single cell suspensions from the lungs (Fig. 6F), indicative of elevated viral titers. CD8^+^ T cells isolated from the mediastinal lymph nodes of these mice, which were cultured *in vitro* with αCD3 for 24 hours, revealed a defect in the ability of these cells from SAA3−/− mice to secrete IFNγ upon restimulation (Fig. 6G). Finally, serum analysis indicated diminished levels of PR8-specific IgG1 and IgG2a in the SAA3−/− mice compared to their wild type littermates (Fig. 6H).

## Discussion

Whereas elevated circulating SAA concentrations have long been correlated with increased innate and adaptive immune responses, the manner in which SAA participates as a mediator of immunity have remained elusive. SAA has been implicated as a pro-inflammatory mediator, but many of the supportive data have been generated through the use of a recombinant protein we have recently reported to contain bacterial lipoproteins^11^. In accordance with our findings, several studies have demonstrated that endogenous SAA does not induce pro-inflammatory responses^31^. There is no debate that levels of SAA are elevated in the setting of inflammatory conditions, therefore it seems unlikely that the process of evolution would afford a mechanism of SAA induction without it having a beneficial effect disproportionally advantageous relative to the expense on fitness experienced as a consequence of its induction. Consequently, it must be participating in a functional manner at some level. Our observations in the setting of SAA3 deficiency indicate that the production of this protein in the setting of inflammation processes may be to modulate cellular innate and adaptive immunity. SAA proteins have been implicated as facilitators of the ability of lipophilic molecules to signal. Included amongst these molecules is retinoic acid in the case of SAA3^17^, and lipoproteins in the case of human SAA1^11^. The absence of SAA3 may seem to have little relevance to the human situation in which SAA3 is an unexpressed pseudogene, and the fact that SAA3 is expressed in non-human organisms at locations more relevant to SAA1 and SAA2.

We have recently published that mice lacking SAA3 exhibit marked alterations in weight gain and innate immunometabolic homeostasis^10^, indicating a profound effect of SAA3 upon normal developmental processes. Consistent with this notion, our results presented herein indicate that mice lacking SAA3 express higher levels of genes associated with fibrosis, exhibit increased levels of high molecular weight citrullinated proteins, and develop intrinsic airway hyperresponsiveness. Citrullination, also known as peptidyl-arginine deamination, is the enzymatic post-translational modification of peptidyl-arginine residues to peptidyl-citrulline^32^. Increases in citrullinated lung proteins have been linked to fibrotic and autoimmune disorders^33^. Citrullinated proteins can drive apoptosis^34^, undergo autophagic processing leading to antigenic presentation by antigen presenting cells^35^, contribute to the pathology of fibrotic disease^36^, and, in the case of rheumatoid arthritis, drive the development of antibodies directed against these self-proteins^37^. In the lung, vimentin, filaggrin, and high-molecular weight cytokeratins are some of the known targets for citrullination^38,39^. The increases in citrullinated proteins present in our SAA3−/− mice may contribute to a pro-fibrotic, pro-autoimmune environment in these animals and, along with the obesity present in these animals, may promote inherent asthma.

The immunometabolic dysfunction observed in these mice included the depressed ability of bone marrow-derived dendritic cells (BMDC) to respond to retinoic acid and to develop immune tolerance to endotoxin^10^. As we report here, SAA3−/− mice exhibit increases in the innate immune cytokines IL-6 and TNFα specifically in the lung, as well as exacerbated inflammatory responses to inhaled LPS. Moreover, intrinsic adaptive responses independent of dendritic cell antigen presentation are altered, as evidenced by the impaired polyclonal stimulation of naïve CD4^+^ and CD8^+^ T cells. Both cell types have repressed basal respiratory and glycolytic function in SAA3−/− mice. CD8^+^ T cells lacking SAA3 produce less IL-2 and IFNγ, and CD4^+^ T cells from SAA3 deficient mice produce increased levels of IL-17A, and significantly decreased levels of T_H_1/T_H_2 cytokines. Although we have reported the robust expression of *Saa3* in the lungs of mice exposed to inhalational regimens that elicit the development of allergic airway disease accompanied by strong T_H_2 and T_H_17 immune responses^9^, exposure of SAA3−/− mice to an allergic asthma model through the instillation of HDM extract revealed no difference in their responses compared to those from wild type mice. It is likely that the intrinsic AHR evident in the SAA3−/− mice obscures their response to methacholine after HDM challenge, and differences between wild type and SAA3−/− mice are not apparent in this robust, acute model of asthma. However, it may be that a chronic model of asthma, in which the effects of the already apparent remodeling that manifest in these mice are present, could reveal a function for SAA3 in disease resolution.

A contribution of SAA3 to disease resolution does appear to be present in our influenza model. Lack of SAA3 led to an increase in mortality in mice that were infected with influenza A virus, as well as increased BAL cell recruitment and viral RNA copies in the lung. Additionally, CD8^+^ T cells from SAA3−/− mice that were restimulated with αCD3 failed to secrete IFNγ. The contribution of IFNγ in H1N1 influenza infection remains controversial, with some data suggesting a role for IFNγ in CD8^+^ T cell homeostasis^27,28^, while others indicate that IFNγ signaling blockade does not alter infection resolution^40,41^. The reasons for this dichotomy remain uncertain, but it has been speculated that the dose and strain of influenza, as well as the timing of IFNγ secretion, may determine the impact of IFNγ during influenza infection. However, not only do we observe a lack of cytokine secretion from CD8^+^ T cells of flu-infected SAA3−/− mice, but also diminished IL-2 and IFNγ secretion from naïve SAA3−/− CD8^+^ cells that have been polyclonally stimulated. This may indicate a basal dysfunction in CD8^+^ T cell activation or increased CD8^+^ T cell apoptosis in SAA3−/− mice, rather than reveal a role for IFNγ signaling specifically in H1N1 influenza. In addition, there is a critical synergy between influenza-specific CD8^+^ T cells and non-neutralizing antibodies that is required for robust immunity to the virus^42^, suggesting a link between the CD8^+^ T cell dysfunction observed in the SAA3−/− mice and their poor ability to generate flu-specific IgG1 and IgG2a. Further studies into the function of SAA3 in the activation, expansion, and survival of antigen-specific CD8^+^ T cells in other infectious models are required to elucidate the means through which SAA3 modulates the immunometabolism of this critical cell population.

Obesity is a risk factor for influenza-induced mortality, and this appears to be in part related to T cell dysfunction. Not only do T cells from obese patients fail to resolve influenza infection, but influenza vaccination is also less efficacious in obese patients, with a decrease in vaccine-induced T memory cells compared to lean individuals; an affect that is recapitulated in diet-induced obese mice^23,43^. This may be due in part to the T_H_17 subset of CD4^+^ T cells, as IL-17A levels are directly correlated to acute lung injury in influenza^24^, and mice on HFD develop increases in IL-17A^+^CD4^+^ αβ^+^ and γδ^+^ cells^44^. We have demonstrated that CD4^+^ T cells from SAA3−/− mice secrete significantly higher levels of IL-17A in response to polyclonal stimulation, and significantly decreased levels of T_H_2 cytokines, indicating a propensity for T_H_17 polarization in these mice compared to wild type. Recent studies have also revealed the critical role for leptin signaling in the influenza response^45^. High levels of leptin observed in obese patients may drive innate proinflammatory responses, while at the same time negatively impacting T cell responses^46^. We have yet to determine the possible obesity-associated alterations in leptin signaling in the SAA3−/− mice and how this may impact lymphocyte proliferation and activity. It remains a target of interest, considering that the SAA3−/− mice demonstrate increases in innate immune signaling coupled with impaired T cell responses.

That obesity has deleterious effects upon immune responses is not a new observation, but only recently have cell-specific metabolic mechanisms been examined in the context of diseases in the lung. The effects of obesity on leukocytes, specifically macrophages, has been found to contribute to the pathology of asthma^47^, and obese alveolar macrophages have decreased activity of PPARγ, an anti-inflammatory nuclear receptor involved in lipid and glucose metabolism^48^. Likewise, adaptive immune cells have metabolic requirements that can become more demanding during infection, such as CD8^+^ T cells, which undergo a switch from oxidative phosphorylation to aerobic glycolysis upon activation^49^.

Further investigation is required to characterize the full extent of lung remodeling, including specific targets of citrullination, present in SAA3 deficient mice. Specifically, how these modifications alter inhalational tolerance, chronic asthma and asthma resolution, and other autoimmune disease models could elucidate the role of SAAs in promoting lung homeostasis. It remains uncertain whether the impact of SAA3 deficiency on decreased survival in influenza virus infection is a consequence of decreased viral clearance, more efficient viral replication, a dysregulated cytokine expression, or a combination of each of these potential contributors. Ultimately, our data highlight the critical role of metabolic dysfunction in impaired immune responses, and implicates SAA as a key mediator at the crossroads of metabolism and immunity.

## Methods

### Mice

C57BL/6 J mice were purchased from The Jackson Laboratory (Bar Harbor, ME). SAA3−/− mice were generated as recently described^10^ and subsequently bred with C57BL/6 J wild type littermates for > 10 generations. Heterozygotes were bred to obtain wild type, heterozygous, and knockout animals. Wild type and knockout animals were then bred as parallel lines. All animals were maintained on 12 hour light/dark cycle and provided chow (~15% kCal from fat; TestDiet, St. Louis, MO) and water ad libitum in an AALAC-accredited facility. All animal experiments were approved by the University of Vermont’s Institutional Animal Care and Use Committee (protocol #12-018), in accordance with the recommendations in the Guide for the Care and Use of Laboratory Animals of the National Institutes of Health, and efforts were made to minimize suffering. Sodium pentobarbital was administered via intraperitoneal injection for euthanasia. Studies involving potentially hazardous materials or recombinant DNA were approved by the University of Vermont’s Institutional Biosafety Committee (protocol #09-018).

### Intranasal lipopolysaccharide challenge

Wild type and SAA3−/− mice were challenged by intranasal instillation with 100 ng LPS from *E*.*coli* 0111:B4 (Invivogen, San Diego, CA) in 40 μl of saline. Mice were analyzed 24 hours after challenge.

### House dust mite (HDM) sensitization and challenge

Wild type and SAA3−/− mice were sensitized by intranasal instillation of 1 μg (by protein) house dust mite extract (Greer, Lenoir, NC) in 40 μl saline. Two weeks later, mice were challenged for 4 consecutive days with 10 μg HDM and analyzed 24 hours after final challenge. Spleens were dissociated through a 70 μm mesh filter and processed to single cell suspensions for restimulation with HDM for 96 hours and supernatants were collected and analyzed by ELISA.

### Pulmonary function assessment

Mice were anesthetized and mechanically ventilated using the forced oscillation technique as previously described^50,51^. Airway resistance (R_N_), tissue damping (G), and tissue resistance (H) were calculated at baseline and after challenge with incremental doses of aerosolized methacholine (0, 3.125, 12.5, 50, and 100 mg/ml) in saline. Peak values after each dose of methacholine were obtained by choosing the highest subsequent value for each individual mouse and averaging it with the two preceding and two following values (for a total of five peak values).

### RNA isolation and quantitative PCR

Tissues were snap-frozen and pulverized in a mortar and pestle in liquid nitrogen. RNA was isolated from lung using the PrepEase kit from Affymetrix (Cleveland, OH) according to manufacturer’s instructions and cDNA was generated from total RNA using the iScript cDNA synthesis kit (Bio-Rad, Hercules, CA) according to manufacturer’s instructions. For The H1N1 analysis of whole lung viral RNA copies^52^, single-cell suspensions were generated from the lungs of wild type and SAA3−/− mice eight days after H1N1 inoculation. Two million cells were collected for RNA and cDNA preparation. Q-PCR was performed using primer pairs (see Table 1) and SYBR Green Universal Taq Mastermix (Bio-Rad). The levels of genes of interest were normalized to the house-keeping gene glyceraldehyde 3-phosphatase dehydrogenase (*Gapdh*) and relative gene expression was calculated using the ∆∆C_T_ method as previously described^53^.

**Table 1:** Primer sequences for genes analyzed by RT-Q-PCR.

Gene	Name	Forward primer	Reverse primer
*Acta2*	airway smooth muscle actin	TGTGCTGGACTCTGGAGATG	GAAGGAATAGCCACGCTCAG
*Actg*	airway actin gamma	GGATCGGTGGCTCCATTCTG	TGAGGTGTGTACATTTGCCAG
*Adipoq*	adiponectin	TGTTCCTCTTAATCCTGCCCA	CCAACCTGCACAAGTTCCCTT
*Clca1*	calcium-activated chloride channel regulator-1 (Gob5)	AAGCAAACCACTCCCATGAC	TGCGAAAGCATCAACAAGAC
*Col1a1*	collagen 1a1	GAGCGGAGAGTACTGGATCG	GTTCGGGCTGATGTACCAGT
*Il1b*	interleukin-1 beta	GCCCATCCTCTGTGACTCAT	AGGCCACAGGTATTTTGTCG
*Il4*	interleukin-4	CCATATCCACGGATGCGACA	AAGCCCGAAAGAGTCTCTGC
*Il5*	Interleukin-5	ATGGAGATTCCCATGAGCAC	GTCTCTCCTCGCCACACTTC
*Il6*	interleukin-6	CCGGAGAGGAGACTTCACAG	GAGCATTGGAAATTGGGGTA
*Il13*	Interleukin-13	CAGCTCCCTGGTTCTCTCAC	TGAGTCCACAGCTGAGATGC
*IL-17a*	interleukin-17A	CTGCTGAGCCTGGCGGCTAC	GGCGGCACTGAGCTTCCCAG
*Il22*	interleukin-22	TCATCGGGGAGAAACTGTTC	CATGTAGGGCTGGAACCTGT
*Ifnb*	interferon-beta	CCACAGCCCTCTCCATCAACTATAAG	AGCTCTTCAACTGGAGAGCAGTTGAG
*Myh11*	myosin heavy chain 11	CTCTGGCCTCTTCTGTGTGG	TCTTTCTTGCCCTTGTGGGA
*Muc5ac*	mucin 5AC	CCATGCAGAGTCCTCAGAACAA	TTACTGGAAAGGCCCAAGCA
*PR8-PA*	polymerase-A gene of PR8 influenza	GAGCCTATGTGGATGGATTC	TGCAGTTCTGCCAGTACTTG
*Saa1*	serum amyloid A1	CCCAGGAGACACCAGGATG	CCCAGCACAACCTACTGAGC
*Saa2*	serum amyloid A2	CCCAGGAGACACCAGCAG	CCCCAGAGAGCATCTTCAGT
*Saa3*	serum amyloid A3	CAGGATGAAGCCTTCCATTG	CATGACTGGGAACAACAGGA
*Tnfα*	tumor necrosis factor alpha	GAACTGGCAGAAGAGGCACT	AGGGTCTGGGCCATAGAACT

### Bronchoalveolar lavage (BAL)

Anesthetized mice were tracheotomized with an 18-gauge cannula and lavaged with 1 ml DPBS (Life Technologies, Carlsbad, CA). Lavage fluid was centrifuged and cell-free supernatants were snap-frozen for analysis. Cell pellets were resuspended and mounted on slides by cytospin for H&E staining.

### Immunoblot for citrullinated peptides

Lung protein lysates were generated from 5 week old wild type and SAA3−/− mice in RIPA Buffer with 0.1% Triton X-100. 40 μg of total protein was run on a 4–20% Tris-Glycine precast gel (Bio-Rad) and transferred to a nitrocellulose membrane using the iBLOT system (Invitrogen, Carlsbad, CA). Total citrullinated peptides were detected using anti-citrullinated antibody (Abcam, Cambridge, MA) and β-actin was detected as a loading control (Sigma-Aldrich, St. Louis, MO). Blots were developed with Pierce ECL Blotting Substrate (Thermofisher, Rockford, IL) and developed on film.

### Immunohistochemistry

Lungs were inflated and fixed in 10% neutral buffered formalin and 5 μm sections were cut and mounted on slides prior to staining with hematoxylin and eosin, Maisson’s Trichrome, or anti-alpha smooth muscle actin (Sigma Aldrich). Stained tissue was imaged using an EVOS XL microscope (Life Technologies) at 20×. For quantitative scoring of alpha smooth muscle actin, photomicrographs were acquired from several areas per slide, coded, and staining intensities around airways were analyzed by three independent observers using a 4-point scale in which 0 = no staining around the outside of airway; 1 = very thin, discontinuous staining around the airway; 2 = thicker, more continuous staining around airways; 3 = airway surrounded by a thick, mostly continuous band.

### Cytokine analysis

BAL fluid and cell supernatants were analyzed for protein secretion with the following assays: IL-4 and TNFα were measured by ELISA kits from BD Biosciences (San Jose, CA). IL-5, IL-10, IL-13, IL-17A, and IFNγ were analyzed by ELISA kits from R&D Technologies (Minneapolis, MN). IL-2 was measured using antibody sets from BD Biosciences. SAA3 was measured either by ELISA kit (Millipore, Billerica, MA) according to manufacturer’s instructions or by Milliplex assay (Millipore).

### Total protein and lactate dehydrogenase analyses

Total protein levels from BAL fluid were measured using the Bradford Assay (Bio-Rad), and lactate dehydrogenase (LDH) activity was measured using the LDH Detection Assay Kit (Promega, Madison, WI).

### CD4^+^ and CD8^+^ T cell analysis

Spleens and lymph nodes (mediastinal and inguinal) were collected from wild type or SAA3−/− mice and processed to single cell suspensions as previously described^54^. Briefly, tissues were dissociated through a 70 μm mesh filter, and white blood cells were isolated using Lymphocyte Separation Media (MP Biomedicals, Solon, OH) according to manufacturer’s protocols. CD4^+^ and CD8^+^ T cells were purified using a magnetic mouse CD4^+^ Negative Selection kit or mouse CD8^+^ Negative Selection kit (Stemcell Technologies, Vancouver, BC) and plated atop 5 µg/ml immobilized anti-CD3 (BD Biosciences) and 4 µg/ml soluble anti-CD28 (BD Biosciences). In some experiments, cells were stained for cell surface markers and intracellular IL-17A. Briefly, polyclonally stimulated CD4^+^ T cells were treated for 4 h before staining with 25 ng/ml phorbol myristate acetate (PMA) and 500 ng/ml Ionomycin in the presence of GolgiPlug (BD Biosciences). Surface staining was performed in FACS Buffer (2% FBS, 0.1% sodium azide in DPBS). Cells were fixed for 1 hour in 4% paraformaldehyde, then permeabilized with 1% FBS, 0.1% saponin, 0.1% sodium azide in DPBS before staining with the intracellular cytokine antibody. Anti-CD4-APC-Cy7, anti-TCRβ-APC, anti-IL-17A-PECF594 were all purchased from BD Bioscience. Anti-TCRδ-BV421 was purchased from Biolegend (San Diego, CA). CD1d tetramer loaded with PBS-57 was provided by the National Institutes of Health (NIH) tetramer facility (Emory University Vaccine Center, Atlanta, GA). Samples were analyzed on the Becton Dickenson LSR II to distinguish as many as 7 fluorophores, and flow data were analyzed using FlowJo Software (Treestar, Ashland, OR). The gating scheme is described in Supplemental Fig. 1.

### H1N1 infection model

Ten to twelve week old wild type and SAA3−/− male littermates were intranasally inoculated with 5000 EIU of H1N1 Puerto Rico/8/34 (PR8) influenza A virus (Charles River Laboratories, Wilmington, MA) in DPBS. Mortality studies were carried out over the course of 22 days, with mice monitored daily and declared moribund once they demonstrated labored breathing, little movement when startled, and weight loss in excess of 30% of their initial weight. In a separate study, mice were inoculated as above and euthanized on Day 8 for analysis of BAL cellularity, lung PR8 RNA copy number^52^, isolation of CD8^+^ cells from the mediastinal lymph node, and measurement of influenza virus A-specific serum immunoglobulin production.

### Serum immunoglobulin quantitation

Flu-specific IgG1 and IgG2a were analyzed by coating 96-well plates with UV-inactivated PR8 H1N1 at 10^7^ EIU/ml and incubating serum on the plate overnight. Biotinylated antibodies for mouse IgG1 and IgG2a (BD Biosciences) were utilized for streptavidin-HRP/TMB substrate ELISA quantitation.

### Analysis of glycolytic and oxidative flux

Real-time changes in extracellular acidification rates (ECARs, as a measure of lactate production) and oxygen consumption rates (OCRs, as a measure of mitochondrial respiration) were analyzed using an XF-96 Extracellular Flux Analyzer (Seahorse Bioscience, North Billerica, MA) as described previously^55^. Polyclonally-stimulated CD4^+^ and CD8^+^ cells were seeded at a density of 4 × 10^5^ cells per well and were analyzed per the manufacturer’s instructions to obtain real-time measurements of baseline OCRs and ECARs.

### Statistics

Data were analyzed by two-tailed unpaired t-test or one-way or two-way ANOVA and Bonferroni post-hoc test using GraphPad Prism 7.04 for Windows (GraphPad Software, Inc, La Jolla, CA). A p value smaller than 0.05 was considered statistically significant.

## Electronic supplementary material

Supplemental Figure 1

## Data Availability

The datasets generated during and/or analysed during the current study are available from the corresponding author on reasonable request.

## References

[1] Levin M, Pras M, Franklin EC (1973). Immunologic studies of the major nonimmunoglobulin protein of amyloid. I. Identification and partial characterization of a related serum component. J Exp Med.

[2] Gorevic PD, Franklin EC (1981). Amyloidosis. Annu. Rev. Med..

[3] Benditt EP, Eriksen N (1977). Amyloid protein SAA is associated with high density lipoprotein from human serum. Proc Natl Acad Sci USA.

[4] Morrow JF, Stearman RS, Peltzman CG, Potter DA (1981). Induction of hepatic synthesis of serum amyloid A protein and actin. Proc Natl Acad Sci USA.

[5] Uhlar CM, Whitehead AS (1999). Serum amyloid A, the major vertebrate acute-phase reactant. Eur J Biochem.

[6] Yamamoto K (1987). Structural diversity of murine serum amyloid A genes. Evolutionary implications. J Immunol.

[7] Steel DM (1993). A constitutively expressed serum amyloid A protein gene (SAA4) is closely linked to, and shares structural similarities with, an acute-phase serum amyloid A protein gene (SAA2). Genomics.

[8] Ramadori G, Sipe JD, Colten HR (1985). Expression and regulation of the murine serum amyloid A (SAA) gene in extrahepatic sites. J Immunol.

[9] Ather JL (2011). Serum amyloid A activates the NLRP3 inflammasome and promotes Th17 allergic asthma in mice. J Immunol.

[10] Ather JL, Poynter ME (2018). Serum amyloid A3 is required for normal weight and immunometabolic function in mice. PLoS One.

[11] Burgess Edward J., Hoyt Laura R., Randall Matthew J., Mank Madeleine M., Bivona Joseph J., Eisenhauer Philip L., Botten Jason W., Ballif Bryan A., Lam Ying-Wai, Wargo Matthew J., Boyson Jonathan E., Ather Jennifer L., Poynter Matthew E. (2018). Bacterial Lipoproteins Constitute the TLR2-Stimulating Activity of Serum Amyloid A. The Journal of Immunology.

[12] Shah C, Hari-Dass R, Raynes JG (2006). Serum amyloid A is an innate immune opsonin for Gram-negative bacteria. Blood.

[13] Hari-Dass R, Shah C, Meyer DJ, Raynes JG (2005). Serum amyloid A protein binds to outer membrane protein A of gram-negative bacteria. J Biol Chem.

[14] Cai Z (2007). Human serum amyloid A protein inhibits hepatitis C virus entry into cells. J Virol.

[15] Lavie M (2006). Serum amyloid A has antiviral activity against hepatitis C virus by inhibiting virus entry in a cell culture system. Hepatology.

[16] Misse D (2007). IL-22 participates in an innate anti-HIV-1 host-resistance network through acute-phase protein induction. J Immunol.

[17] Derebe MG (2014). Serum amyloid A is a retinol binding protein that transports retinol during bacterial infection. Elife.

[18] Chen F (2014). Prenatal retinoid deficiency leads to airway hyperresponsiveness in adult mice. J Clin Invest.

[19] Hall JA, Grainger JR, Spencer SP, Belkaid Y (2011). The role of retinoic acid in tolerance and immunity. Immunity.

[20] McGowan SE, Holmes AJ, Smith J (2004). Retinoic acid reverses the airway hyperresponsiveness but not the parenchymal defect that is associated with vitamin A deficiency. Am J Physiol Lung Cell Mol Physiol.

[21] Ather Jennifer L, Poynter Matthew E, Dixon Anne E (2015). Immunological characteristics and management considerations in obese patients with asthma. Expert Review of Clinical Immunology.

[22] Shore Stephanie A. (2008). Obesity and asthma: Possible mechanisms. Journal of Allergy and Clinical Immunology.

[23] Sheridan PA (2012). Obesity is associated with impaired immune response to influenza vaccination in humans. Int J Obes (Lond).

[24] Bermejo-Martin JF (2009). Th1 and Th17 hypercytokinemia as early host response signature in severe pandemic influenza. Crit Care.

[25] Li C (2012). IL-17 response mediates acute lung injury induced by the 2009 pandemic influenza A (H1N1) virus. Cell Res.

[26] Crowe CR (2009). Critical role of IL-17RA in immunopathology of influenza infection. J Immunol.

[27] Bot A, Bot S, Bona CA (1998). Protective role of gamma interferon during the recall response to influenza virus. J Virol.

[28] Turner SJ, Olivas E, Gutierrez A, Diaz G, Doherty PC (2007). Disregulated influenza A virus-specific CD8 + T cell homeostasis in the absence of IFN-gamma signaling. J Immunol.

[29] Wilson TC, Bachurski CJ, Ikegami M, Jobe AH, Kallapur SG (2005). Pulmonary and systemic induction of SAA3 after ventilation and endotoxin in preterm lambs. Pediatr Res.

[30] Hiratsuka S (2013). Primary tumours modulate innate immune signalling to create pre-metastatic vascular hyperpermeability foci. Nat Commun.

[31] Christenson K (2013). Endogenous Acute Phase Serum Amyloid A Lacks Pro-Inflammatory Activity, Contrasting the Two Recombinant Variants That Activate Human Neutrophils through Different Receptors. Front Immunol.

[32] van Venrooij WJ, Pruijn GJ (2000). Citrullination: a small change for a protein with great consequences for rheumatoid arthritis. Arthritis Res..

[33] Valesini G (2015). Citrullination and autoimmunity. Autoimmun. Rev..

[34] Inagaki M, Takahara H, Nishi Y, Sugawara K, Sato C (1989). Ca2 + -dependent deimination-induced disassembly of intermediate filaments involves specific modification of the amino-terminal head domain. J Biol Chem.

[35] Ireland JM, Unanue ER (2012). Processing of proteins in autophagy vesicles of antigen-presenting cells generates citrullinated peptides recognized by the immune system. Autophagy.

[36] Vassiliadis E (2012). Circulating levels of citrullinated and MMP-degraded vimentin (VICM) in liver fibrosis related pathology. Am J Transl Res.

[37] Szodoray P (2010). Anti-citrullinated protein/peptide autoantibodies in association with genetic and environmental factors as indicators of disease outcome in rheumatoid arthritis. Autoimmun. Rev..

[38] Catrina AI, Ytterberg AJ, Reynisdottir G, Malmstrom V, Klareskog L (2014). Lungs, joints and immunity against citrullinated proteins in rheumatoid arthritis. Nat. Rev. Rheumatol..

[39] Hutchinson D (2018). Cadmium nanoparticles citrullinate cytokeratins within lung epithelial cells: cadmium as a potential cause of citrullination in chronic obstructive pulmonary disease. Int. J. Chron. Obstruct. Pulmon. Dis..

[40] Price GE, Gaszewska-Mastarlarz A, Moskophidis D (2000). The role of alpha/beta and gamma interferons in development of immunity to influenza A virus in mice. J Virol.

[41] Prabhu N (2013). Gamma interferon regulates contraction of the influenza virus-specific CD8 T cell response and limits the size of the memory population. J Virol.

[42] Laidlaw BJ (2013). Cooperativity between CD8 + T cells, non-neutralizing antibodies, and alveolar macrophages is important for heterosubtypic influenza virus immunity. PLoS Pathog.

[43] Karlsson EA, Sheridan PA, Beck MA (2010). Diet-induced obesity impairs the T cell memory response to influenza virus infection. J Immunol.

[44] Mathews JA, Wurmbrand AP, Ribeiro L, Neto FL, Shore SA (2014). Induction of IL-17A Precedes Development of Airway Hyperresponsiveness during Diet-Induced Obesity and Correlates with Complement Factor D. Front Immunol.

[45] Radigan KA (2014). Impaired clearance of influenza A virus in obese, leptin receptor deficient mice is independent of leptin signaling in the lung epithelium and macrophages. PLoS One.

[46] Papathanassoglou E (2006). Leptin receptor expression and signaling in lymphocytes: kinetics during lymphocyte activation, role in lymphocyte survival, and response to high fat diet in mice. J Immunol.

[47] Lugogo NL (2012). Alveolar macrophages from overweight/obese subjects with asthma demonstrate a proinflammatory phenotype. Am J Respir Crit Care Med.

[48] Sharma S (2012). Alveolar macrophage activation in obese patients with obstructive sleep apnea. Surgery.

[49] Palmer CS, Ostrowski M, Balderson B, Christian N, Crowe SM (2015). Glucose metabolism regulates T cell activation, differentiation, and functions. Front Immunol.

[50] Poynter ME (2004). NF-kappa B activation in airways modulates allergic inflammation but not hyperresponsiveness. J Immunol.

[51] Ather JL, Hodgkins SR, Janssen-Heininger YM, Poynter ME (2011). Airway epithelial NF-kappaB activation promotes allergic sensitization to an innocuous inhaled antigen. Am J Respir Cell Mol Biol.

[52] Jelley-Gibbs DM (2007). Persistent depots of influenza antigen fail to induce a cytotoxic CD8 T cell response. J Immunol.

[53] Bevelander M (2007). Nitrogen dioxide promotes allergic sensitization to inhaled antigen. J Immunol.

[54] Hodgkins SR (2010). NO2 inhalation induces maturation of pulmonary CD11c + cells that promote antigenspecific CD4 + T cell polarization. Respir. Res..

[55] Amiel E (2014). Mechanistic target of rapamycin inhibition extends cellular lifespan in dendritic cells by preserving mitochondrial function. J Immunol.

