# Supplementary material for: Serum Amyloid A3 is required for normal lung development and survival following influenza infection
Source: Sci Rep. 2018 Nov 8;8:16571. doi: 10.1038/s41598-018-34901-x (PMC6224415; doi:10.1038/s41598-018-34901-x)
Supplement: Supplementary file 1 — Supplemental Figure 1 [file 41598_2018_34901_MOESM1_ESM.pdf]

Serum Amyloid A3 is required for normal lung development and survival following influenza infection

Jennifer L. Ather[1], Oliver Dienz[2], Jonathan E. Boyson[2], Vikas Anathy[3], Eyal Amiel[4], and Matthew E. Poynter[1*]

Vermont Lung Center, Division of Pulmonary Disease and Critical Care, Department of Medicine[1], Department of Surgery[2], Department of Pathology and Laboratory Medicine[3], and Department of Biomedical and Health Sciences[4], University of Vermont, Burlington, VT 05405

*Corresponding author: Matthew E. Poynter
University of Vermont
Given E410A, 89 Beaumont Ave.
Burlington, VT 05405
802-656-8045
Matthew.Poynter@uvm.edu

Supplemental Figure 1

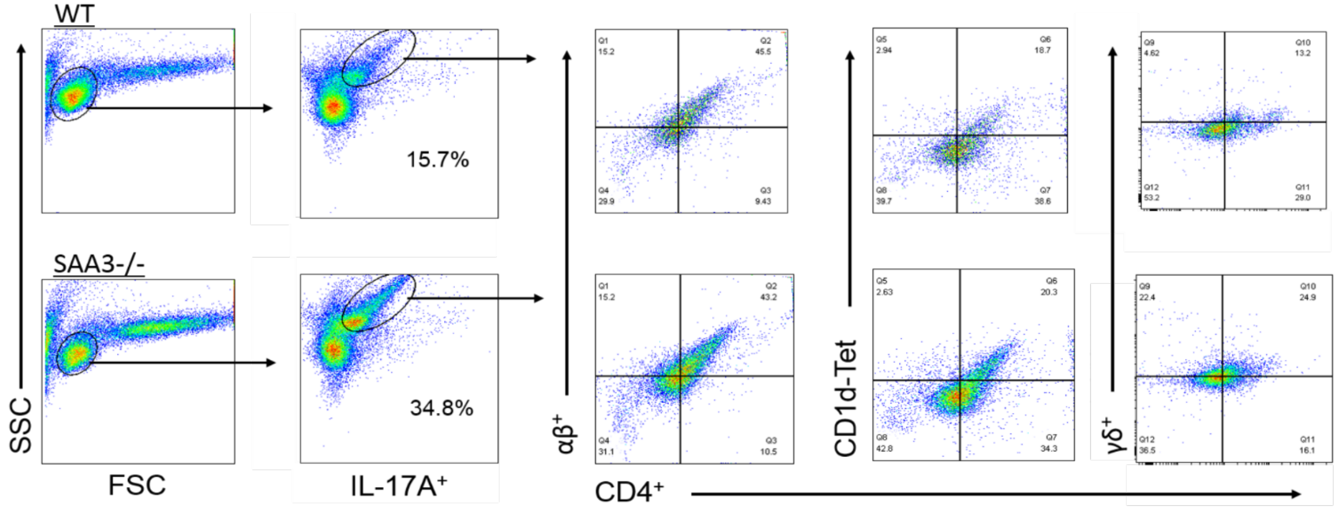

Supplemental Figure 1. Gating scheme for flow cytometry analysis of cultured CD4$^+$ T cells from wild type and SAA3-/- mice. Following 96-hour polyclonal stimulation with αCD3/αCD28, CD4$^+$ T cells were collected and analyzed by flow cytometry. Cells were first gated on a FSC/SSC-Lo population that corresponds to lymphocyte size and granularity. Lymphocytes were then gated upon intracellular expression of IL-17A. The lymphocytes IL-17A$^+$ populations were then characterized for co-expression of CD4 with TCRαβ, TCRγδ, or CD1d-tetramer (an NKT cell marker).
